# Stability of MDS-UPDRS Motor Subtypes Over Three Years in Early Parkinson's Disease

**DOI:** 10.3389/fneur.2021.704906

**Published:** 2021-09-24

**Authors:** Abhijeet K. Kohat, Samuel Y. E. Ng, Aidan S. Y. Wong, Nicole S. Y. Chia, Xinyi Choi, Dede L. Heng, Wei Li, Hwee-Lan Ng, Shu-Ting Chua, Shermyn X. M. Neo, Zheyu Xu, Kay-Yaw Tay, Wing-Lok Au, Eng-King Tan, Louis C. S. Tan

**Affiliations:** ^1^Department of Neurology, National Neuroscience Institute, Singapore, Singapore; ^2^Parkinson's Disease and Movement Disorders Centre (Parkinson Foundation's International Center of Excellence), National Neuroscience Institute, Singapore, Singapore; ^3^Duke-NUS Graduate Medical School, Singapore, Singapore

**Keywords:** subtype, parkinson's disease, tremor dominant (TD), postural instability and gait difficulties, indeterminate, over time, stability

## Abstract

**Background:** Various classifications have been proposed to subtype Parkinson's disease (PD) based on their motor phenotypes. However, the stability of these subtypes has not been properly evaluated.

**Objective:** The goal of this study was to understand the distribution of PD motor subtypes, their stability over time, and baseline factors that predicted subtype stability.

**Methods:** Participants (*n* = 170) from two prospective cohorts were included: the Early PD Longitudinal Singapore (PALS) study and the National Neuroscience Institute Movement Disorders Database. Early PD patients were classified into tremor-dominant (TD), postural instability and gait difficulty (PIGD), and indeterminate subtypes according to the Movement Disorder Society's Unified PD Rating Scale (MDS-UPDRS) criteria and clinically evaluated for three consecutive years.

**Results:** At baseline, 60.6% patients were TD, 12.4% patients were indeterminate, and 27.1% patients were PIGD subtypes (*p* < 0.05). After 3 years, only 62% of patients in TD and 50% of patients in PIGD subtypes remained stable. The mean levodopa equivalent daily dose (LEDD) was higher in the PIGD subtype (276.92 ± 232.91 mg; *p* = 0.01). Lower LEDD [*p* < 0.05, odds ratio (OR) 0.99, 95% confidence interval (CI): 0.98–0.99] and higher TD/PIGD ratios (*p* < 0.05, OR 1.77, 95% CI: 1.29–2.43) were independent predictors of stability of TD subtype with an area under the curve (AUC) of 0.787 (95%CI: 0.669–0.876), sensitivity = 57.8%, and specificity = 89.7%.

**Conclusion:** Only 50–62% of PD motor subtypes as defined by MDS-UPDRS remained stable over 3 years. TD/PIGD ratio and baseline LEDD were independent predictors for TD subtype stability over 3 years.

## Introduction

More than 6 million people suffer from Parkinson's disease (PD) worldwide, and these numbers will continue to rise due to increasing life expectancy and aging (1, 2). The pathology first begins in non-dopaminergic neurons, resulting in appearance of non-motor symptoms (NMS) at early courses of the disease (3). The diagnosis of clinical PD relies on core motor symptoms, while prodromal PD can be defined largely by non-motor features (4). Research have found a significant correlation between NMS burden and PD motor subtypes (5). Various classification schemes have consequently merged NMS and motor features to classify PD subtypes (6–8). Fereshtehnejad et al. (9) included cognitive impairment, rapid eye movement sleep behavior disorder, and dysautonomia for the classification of PD subtypes. Furthermore, a cluster analysis further identified various clusters of PD by combining motor and NMS. However, PD patients show striking differences in the progression of the disease and disease-specific symptoms, which are ever-changing with disease progression. Due to this heterogeneity, PD patients have been grouped according to the dominance of the symptoms. Recognition of these subgroups is important as it may define disease prognosis and impact clinical trial designs.

At present, there are no consensus on the methodology of PD subtyping for research or clinical practice. Since the early 1980s, PD subtypes have been classified by various methods with motor subtypes taking dominance. Two motor subtypes have been well-established in literature: tremor predominant and akinetic–rigid (AR) (10, 11). With the development of the Unified Parkinson's Disease Rating Scale (UPDRS), PD motor subtypes could be defined more objectively. Other than tremor-dominant (TD) and postural instability and gait difficulty (PIGD) subtypes, axial-dominant (AxD), appendicular-dominant (ApD), and rigidity-dominant (RD) subtypes were also used to classify PD subtypes (11–15). However, TD and PIGD remain the dominantly studied subtypes in literature. ApD and RD subtypes remain ill-defined in the literature, and clinicians prefer to classify these subtypes under the indeterminate type. Eisinger et al. further defined these subtypes according to AxD subscore/(ApD subscore + RD subscore) ratio. Total ratio scores of more than 1 was suggestive of the AxD subtype. Similarly, ApD subscore/RD subscore > 1 favors ApD subtypes and <1 suggested RD subtype (15). In the method of Kang et al., the ratio of the mean UPDRS tremor scores (UPDRS III items 20–21 divided by 4) to the mean UPDRS AR scores (UPDRS III items 22–27 and 31 divided by 15) was used to identify TD (ratio > 1), mixed (0.8–1.0), and AR subtypes (ratio <0.8) (14). Konno et al. in a retrospective analysis of 1,003 PD patients classified the PD subtypes according to the predominant clinical features. If rigidity and bradykinesia were featured prominently, then the patient was classified as having AR subtype. If gait difficulty was the prominent abnormality, then the patient was classified as gait difficulty subtype (16). Choi et al. (17) grouped 192 *de novo* PD patients in TD/mixed/AR and TD/mixed/PIGD subtypes according to two methods proposed by Jankovic et al. and Kang et al. With the development of the Movement Disorder Society's Unified PD Rating Scale (MDS-UPDRS), Stebbins et al. further validated these subtypes with optimal specificity and sensitivity obtained for the TD/PIGD ratio to define TD, indeterminate, and PIGD subtypes (18).

To date, only one previous study has investigated motor subtypes using the criteria set by Stebbins et al. However, one limitation of that study was that the study had a short follow-up duration of 12 months (19). Another recent study studied the various subtyping methods and their stability, but had a relatively short follow-up period of 1 month (20). We, therefore, undertook this study to understand (1) the distribution of pre-defined motor subtypes of PD using the MDS-UPDRS criteria, (2) their stability over 3 years, and (3) determine independent factors predicting the stability of these subtypes.

## Materials and Methods

### Study Settings

This is a retrospective study based on data collected prospectively from idiopathic PD patients from the Early PD Longitudinal Singapore (PALS) study and the National Neuroscience Institute Movement Disorders Database. The PALS study is a prospective cohort study undertaken to understand PD progression, while the Movement Disorders Database has prospectively collected clinical information from a clinic cohort and has been in existence since 2002. Details of both cohorts have been described previously (21, 22). The clinical diagnosis was made by movement disorder specialists and adhered to the diagnostic criteria defined by the National Institute of Neurological Disorders and Stroke (NINDS) for PD (23). The study was approved by the Centralized Institutional Review Board of the Singapore Health Services.

### Inclusion Criteria

All the participants were examined by movement disorder specialists at the National Neuroscience Institute, a tertiary center in Singapore, and evaluated within 2 years from their diagnosis at the institute. MDS-UPDRS was performed annually for three consecutive years when on medications from their first clinical visit. Each patient was evaluated and followed by the same neurologist on each visit. The institute has adopted the MDS-UPDRS for clinical evaluation of PD patients since 2014 after all clinicians had completed and passed the training program.

### Study Procedures

The MDS-UPDRS at baseline and subsequent visits was completed in the on-medication state. Levodopa equivalent daily dose (LEDD; mg/day) at baseline evaluation was calculated from the dosages of prescribed dopaminergic drugs based on the standard conversion table as previously reported (24). Patients were categorized into Parkinson's subtypes based on the MDS-UPDRS according to the criteria set by Stebbins et al. (18). Patients were subtyped into three categories—TD, indeterminate, and PIGD—according to the TD/PIGD ratio. TD score was calculated by adding 11 items in the MDS-UPDRS (2.10 and 3.15–3.18). PIGD score was calculated by adding five items in the MDS-UPDRS (2.12. Walking and balance, 2.13. Freezing, 3.10. Gait, 3.11. Freezing of gait, and 3.12. Postural stability). TD vs. PIGD subtype is calculated as a ratio of the tremor vs. PIGD mean scores. Cut-off levels for the MDS-UPDRS TD subtype is a TD/PIGD ratio of >1.15 and for PIGD subtype is <0.90. Subjects with ratios between 0.9 and 1.15 are classified as indeterminate. Cognition was evaluated at baseline and then annually using the Mini-Mental State Examination (MMSE).

### Definition of Stability

**Figure 1:**
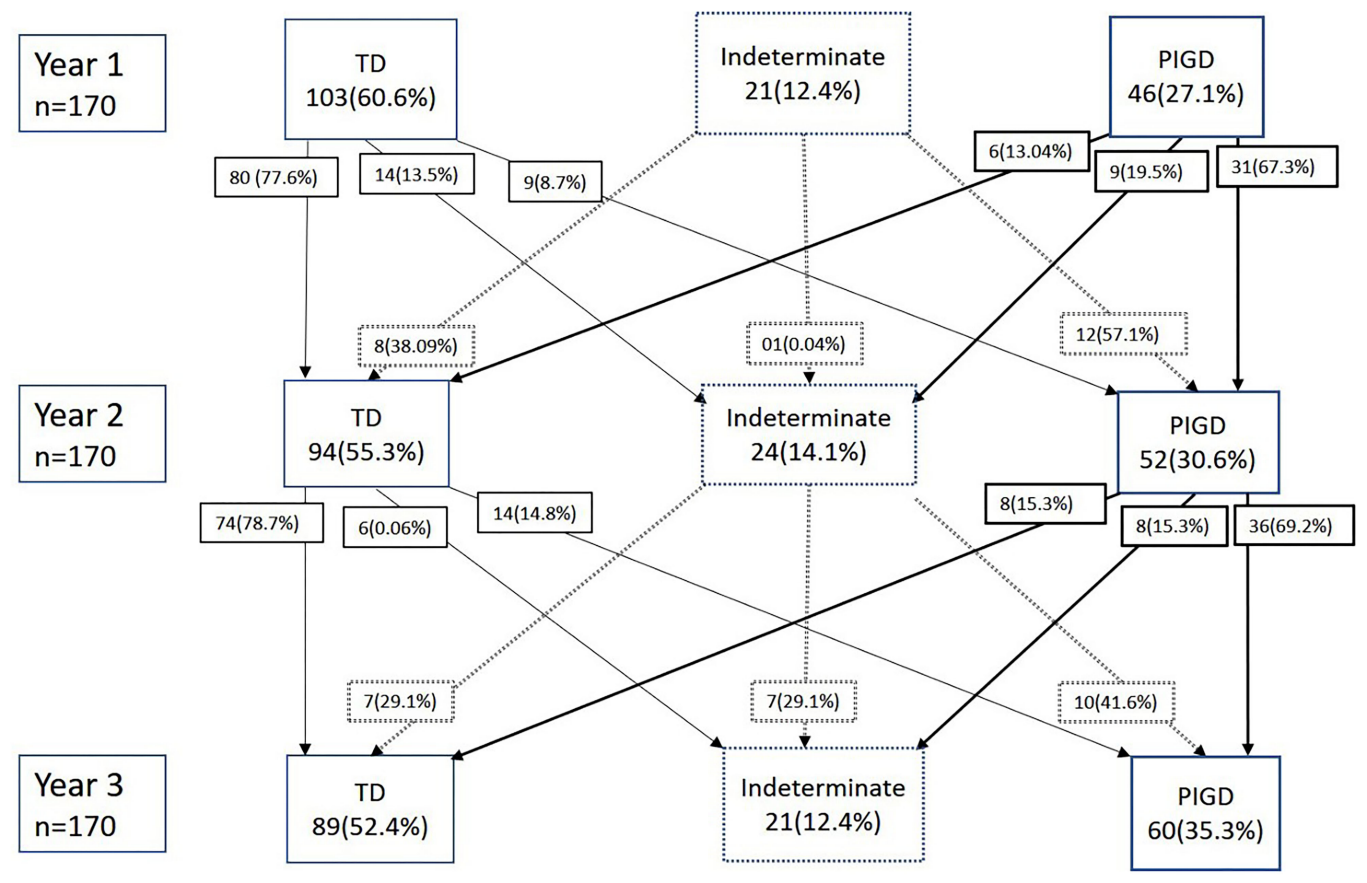
Shifts in Parkinson's disease (PD) subtypes over 3 years.

Participants were deemed to have a “stable” subtype if their subtype remains the same throughout all 3 years and an “unstable” subtype if their subtype classification shifts at any point during the follow-up (Figure 1).

This study aims to understand the distribution of pre-defined motor subtypes of PD using the MDS-UPDRS criteria and to examine the stability of Parkinson's subtype during the follow-up of 3 years in patients with early PD. We also sought to identify the independent predictors of stability of the subtypes that included age, sex, LEDD, and TD/PIGD ratio.

### Statistical Analysis

Descriptive statistics including mean ± standard deviation and median (interquartile) and frequency (%) is reported for continuous variables and categorical variables, respectively. The baseline characteristics of the patients were compared among the subtypes using analysis of variance (ANOVA) or Kruskal-Wallis test for continuous variables (depending whether the normality assumption on the residuals was tenable) and chi-square test for categorical variables. ANOVA was performed for age, gender, disease duration, MDS-UPDRS III, and MMSE; Kruskal-Wallis was conducted for LEDD; and chi-square for gender and subtype stability, respectively. *Post-hoc* analyses were performed using Bonferroni and Dunn's test, respectively, for significant variables. Multivariable logistic regression analysis was used to investigate the association of the baseline variables in TD and PIGD subtypes and the stability of subtypes (as the binary outcome variable), and odds ratio (OR) along with 95% confidence interval (CI) were reported. Specificity and sensitivity of factors predicting the TD subtype were analyzed from the area under the curve (AUC) using receiver operating characteristics (ROC). Youden index (sensitivity + specificity−1) approach was used to calculate the optimal cut-off point in the ROC analysis. Significance level was set at a *p* < 0.05. The statistical analysis was performed in SPSS (IBM Corp. Released 2013. IBM SPSS Statistics for Windows, Version 22.0. Armonk, NY: IBM Corp.).

## Results

A total of 170 idiopathic PD patients that comprised of 111 patients from the PALS cohort and 59 from the Database cohort were included in the study. There were 100 (58.8%) out of 170 patients who were male. Baseline demographic and clinical characteristics are summarized in Table 1. The mean LEDD mg/day requirement was higher in the PIGD subtype (276.92 ± 232.91 mg/day) compared to TD (192.75 ± 144.21 mg/day) and indeterminate subtype (257.76 ± 177.72 mg/day; *p* = 0.01). It should be also noted that there is a slightly higher dispersion in LEDD in the PIGD compared to TD or indeterminate. The coefficient of variation of LEDD is 0.75, 0.69, and 0.84 in TD, indeterminate, and PIGD groups, respectively. When baseline MMSE was compared between stable (*n* = 88) and unstable (*n* = 82) groups, no significant differences were found between the two groups (26.45 ± 3.59 vs. 26.88 ± 3.04, *p* = 0.41).

**Table 1:** Baseline variables of subtypes and their stability at 3 years.

**Variable**	**All**	**TD**	**Indeterminate**	**PIGD**	Statistics^*^	***p* ^+^**
	**(*n* = 170)**	**(*n* = 103)**	**(*n* = 21)**	**(*n* = 46)**		
Age (years)	69.3 ± 9.7	68.8 ± 8.10	73.5 ± 9.42	68.4 ± 12.3	2.35	0.099
Gender (male, %)	100 (58.8)	61 (59.2)	15 (71.4)	24 (52.2)	2.22	0.329
Education (years)	9.55 ± 4.61	9.80 ± 4.26	9.14 ± 5.47	9.19 ± 4.98	0.386	0.693
LEDD, mg	233.56 ± 177.72	192.75 ± 144.21	257.76 ± 177.72	276.92 ± 232.91	6.09	**0.048**
Disease duration (months)	6.35 ± 6.50	6.01 ± 5.89	5.52 ± 5.51	7.49 ± 8.03	1.02	0.361
MMSE	26.66 ± 3.34	26.88 ± 3.12	26.67 ± 2.65	26.15 ± 4.03	0.760	0.469
MDS-UPDRS Part III	23.51 ± 11.36	22.66 ± 11.24	23.09 ± 12.69	25.61 ± 10.98	1.08	0.342
**Stability**, ***N*** **(% of subtypes)**						
Stable		64 (62.1)	1 (4.8)	23 (50.0)	23.1	**<0.001**
Unstable		39 (37.9)	20 (95.2)	23 (50.0)		

*Mean ± standard deviation and median (interquartile) reported for continuous variables; frequency (%) reported for categorical variables*.

+ *ANOVA or Kruskal–Wallis test (depending on the normality assumption) for continuous variables and chi-square for categorical variables; ANOVA was performed for age, gender, disease duration, MDS-UPDRS III, and MMSE; Kruskal–Wallis was conducted for LEDD; and chi-square for gender and stability*.

* *Reported statistics is chi-square for categorical variables and non-normal continuous variables; F value for normal continuous variables; degree of freedom = 2 for all variables as the grouping variable include three categories*.

LEDD, baseline levodopa equivalent dosages; TD, tremor-dominant subtype; PIGD, postural instability gait subtype; MMSE, Mini-Mental State Examination.

*Significant values are indicated in bold*.

*Post-hoc* analysis revealed the LEDD dose to be significantly higher in the PIGD subtype as compared to the TD subtype.

The stability and fluctuations of PD subtypes over the 3 years is summarized in Figure 1. At baseline (year 1), 103 (60.6%) patients were TD, 21 (12.4%) patients were indeterminate, and 46 (27.1%) patients were PIGD subtypes. On the second year of follow-up, 80 (77.6%) of the TD patients remained as TD, whereas 31 (67.3%) of the PIGD remained as PIGD. At the end of 3 years, 62% of patients in TD and 50% of patients in PIGD subtypes were stable and had remained in the same subtype group over 3 years. In the indeterminate subtype, only one patient (4.8%) remained stable. *Post-hoc* analyses revealed that both the TD and PIGD subtypes to both be significantly stable as compared to the indeterminate subtype.

In multivariate analysis, male gender (*p* = 0.04, OR 0.35, 95% CI: 0.12–0.98), lower LEDD (*p* < 0.05, OR 0.99, 95% CI: 0.98–0.99), and higher TD/PIGD ratio (*p* < 0.05, OR 1.77, 95% CI 1.29–2.43) at baseline were the independent predictors of stability over 3 years for the TD subtype. There was no statistically significant predictor found for the PIGD subtype (Table 2). In particular, we found that a more dominant tremor score with higher TD/PIGD ratio had a 77% higher chance of having a stable TD subtype (Table 2). Although we had assessed cognition *via* the MMSE, baseline cognitive scores did not significantly predict the stability of the subtypes.

**Table 2:** Multivariable analysis of baseline variables in predicting subtype stability.

**Baseline variables**	**Univariate**			Multivariable^*^		
	**OR (95% CI)**	***P***	Chi-square^**^	**OR (95% CI)**	***P***	Chi-square^**^
Tremor subtype						36.7
Age, years	1.03 (−1.13, 1.24)	0.178	1.85	1.04 (0.98, 1.11)	0.154	
Gender (female)	0.41 (0.18, 0.91)	**0.037**	4.42	0.35 (0.12, 0.98)	**0.047**	
Education, years	0.98 (0.88, 1.06)	0.176	6.75	0.92 (0.81, 1.05)	0.237	
LEDD, mg	0.99 (0.99, 1.00)	**0.037**	4.52	0.99 (0.98, 0.99)	**0.006**	
TD/PIGD ratio	1.72 (1.30, 2.26)	**<0.001**	23.7	1.77 (1.29, 2.43)	**<0.001**	
Disease duration, years	0.99 (0.92, 1.05)	0.780	0.077	0.98 (0.90, 1.07)	0.766	
MMSE	0.96 (0.84, 1.10)	0.623	0.247	1.01 (0.85, 1.19)	0.892	
PIGD subtype						5.06
Age	1.01 (0.96, 1.06)	0.694	0.159	0.99 (0.93, 1.06)	0.884	
Gender	1.41 (0.44, 4.53)	0.555	0.349	1.92 (0.50, 7.27)	0.337	
Education	1.01 (0.89, 1.13)	0.881	0.022	1.09 (0.93, 1.28)	0.275	
LEDD	1.00 (0.99, 1.00)	0.415	0.704	1.00 (0.99, 1.00)	0.780	
TD/PIGD ratio	0.21 (0.01, 2.83)	0.242	1.40	0.16 (0.01, 2.77)	0.211	
Disease duration	0.99 (0.92, 1.05)	0.780	0.077	0.96 (0.88, 1.06)	0.493	
MMSE	0.91 (0.782, 1.07)	0.300	1.18	0.85 (0.66, 1.09)	0.223	

* *Multivariable logistical regression model. Multivariable model is only provided for tremor-dominant subtype and PIGD subtype*.

** *Chi-square statistics with one degree of freedom is reported for each variable*.

*LEDD, levodopa equivalent daily dose; MMSE, Mini-Mental State Examination; TD, tremor-dominant subtype; PIGD, postural instability gait subtype; OR, odds ratio; CI, confidence interval*.

*Significant values are indicated in bold*.

**Figure 2:**
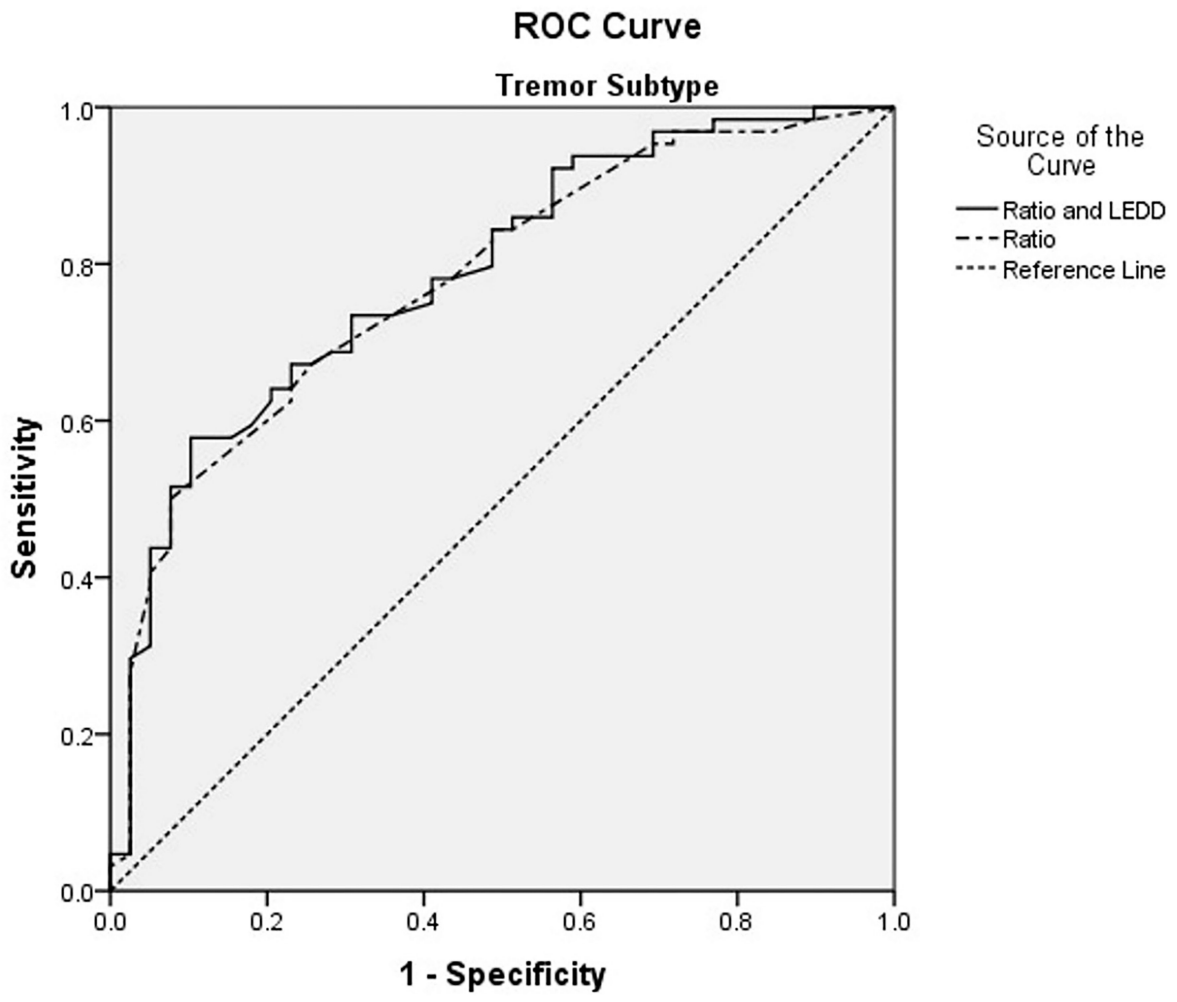
ROC curve of levodopa equivalent daily dose (LEDD) and tremor dominant (TD)/ postural instability and gait difficulty (PIGD) ratio for TD subtype stability.

ROC analysis reviewed utilizing the Youden index (sensitivity + specificity – 1) revealed that higher TD/PIGD ratio scores with a cut-off of 4.2 could predict TD subtype stability over 3 years with an AUC of 0.78, 95% CI (0.69–0.87), sensitivity = 50.0%, and specificity = 99.9% (Table 3, Figure 2). A combination of LEDD and ratio scores with a cut-off LEDD of 229.6 mg and ratio of 4.0 improved prediction of TD subtype stability with an AUC of 0.79, 95% CI (0.67–0.88), sensitivity = 57.8%, and specificity = 89.7%.

**Table 3:** Receiver operating curve (ROC) analysis for subtype stability^*^.

**Indicator**	**AUC (95% CI)**	***p***	**Composite probability cut-off**	**Corresponding LEDD cut-off (mg)**	**Corresponding ratio cut-off**	**Sensitivity (%)**	**Specificity (%)**
TD/PIGD ratio	0.779 (0.689–0.869)	<0.0001		–	4.2	50.0	99.9
LEDD & TD/PIGD ratio	0.787 (0.699–0.876)	<0.0001	0.654	229.6	4.0	57.8	89.7

* *ROC analysis for tremor-dominant subtype*.

## Discussion

In this study from two prospective cohorts, we reported the stability of PD motor subtypes as defined by MDS-UPDRS scores over 3 years. Only 62% of TD and 50% of PIGD patients maintained their subtype stability in the early stages of the disease over 3 years. The indeterminate subtype group was the most unstable subtype of all, possibly due to its fluctuating nature. PIGD patients had higher LEDD. TD/PIGD ratio and LEDD were the independent predictors of the TD subtype stability over 3 years. A combination of both factors after optimization of their cut-offs improved the identification of TD patients who remained stable at 3 years, with a sensitivity and specificity of 58 and 90%, respectively.

There is a scarcity of studies that have investigated the stability of PD motor subtypes. Even among these few studies, the study populations, the study durations, and criteria used to perform motor subtyping have been varied. Schiess et al. (12) modified the original TD/PIGD ratio suggested by Jankovic et al. to subtype PD patients. Patients were classified according to Schiess ratio by applying the UPDRS to define TD (1.01–2.13), AR (0.23–0.80), and mixed (0.78–0.97) subtypes. However, this study only had 30 participants comprising 11 AR and 10 TD patients. Eisinger et al. (15) using the PPMI data identified unique motor subtypes based on the MDS-UPDRS. In addition to the TD and PIGD subtypes, the indeterminate subtype was sub-grouped into AxD, ApD, and RD subtypes. The study found that with increasing disease duration, consistent with the findings of our study, the prevalence of TD subtypes decreased while that of PIGD increased.

The PPMI cohort provided further insights into the motor classification of PD, using the MDS-UPDRS (17, 19). At baseline, 71% of participants were TD and 18% were PIGD subtype. Our results are in agreement with the PPMI study where the majority of the patients belonged to the TD subtype (60% TD and 27% PIGD at baseline assessment). This is in contrast to earlier studies where AR or PIGD subtypes were more prominent when the original UPDRS and its criteria for subtypes were adopted. The reason for this could be the inclusion of additional domains to assess tremors in the MDS-UPDRS (11 vs. 8). At the end of the 1-year follow-up in the PPMI cohort, 18% TD and 39% PIGD had shifted subtypes in the cohort, as compared to 23% of TD and 33% PIGD in our cohort. At the end of 3 years, 38% of TD and 50% of PIGD patients had unstable subtypes and shifted over 3 years. In the PPMI cohort, one-third of the indeterminate subtype patients were stable and two-thirds had their subtypes shift at 1 year. In our study, only one patient was stable in the indeterminate subtype after the third year, and the majority shifted subtypes across the years. Indeterminate subtypes consist of the patients whose PD subtypes are dynamic and whose signs are variable, so they are prone for rapid change in subtypes. Taken together, these findings suggest that the PIGD subtype as defined by the MDS-UPDRS is more unstable than the TD subtype over time, and this may have implications on patient selection for clinical studies and trials that are stratified by such subtypes.

We further explored clinical markers that predicted the stability of PD. The PIGD subtype had significantly higher mean LEDD compared to the other two subtypes. This can be explained by the presence of greater motor deficits in PIGD patients that required more dopaminergic medications (14, 23). After 3 years of follow-up, we found that lower LEDD doses and higher TD/PIGD ratios at baseline were significant predictors for TD patients to remain stable over 3 years. Further ROC analysis showed that when LEDD and higher TD/PIGD ratios were combined, TD stability over 3 years could be predicted with a sensitivity and specificity of 58 and 90%, respectively. Our results strongly suggest that classification of motor subtypes in PD can shift with increasing disease duration. These results provide the basis for subsequent refinement of the MDS-UPDRS subtype criteria to better define and characterize motor subtypes that remain stable over the course of the disease.

We believe that these motor subtypes are clinically and pathophysiologically distinct, and their clinical implications are also different. The TD subtype has a more benign course, comparatively slower rate of progression, and better response to levodopa therapy (24). The PIGD subtype on the other hand has been associated with more disabilities; rapid progression; cognitive decline, especially working memory; and greater association with non-motor features (25–29). TD patients also have a lower risk of developing dementia until they are transformed into the PIGD subtype (30). On imaging, both subtypes showed notable differences on FP-CIT single-photon emission computed tomography (SPECT) with TD patients found to have an “eagle-winged” striatal configuration, suggestive of dopamine loss in the caudate and lateral putamen, whereas patients with PIGD subtype had dopaminergic loss in the dorsal putamen and appeared as an “egg-shaped” configuration (31).

Despite the traditional reliance on motor subtypes to classify PD, the results of this study and previous investigations demonstrate a lack of stability for these motor subtypes over time. This brings into question the reliability and usefulness of the various criteria that have been used to perform motor subtyping. In view of this, PD subtyping has evolved to include data-driven approaches such as cluster analysis (CA), which is a highly sensitive statistical method that has gained favor in recent times. The major advantage is that these subtypes are derived from data rather than from pre-defined criteria used in conventional classification systems and can include additional variables other than conventional motor ones. In a systematic review, seven studies were identified that used CA to identify subtypes in the broad clinical spectrum of PD (25, 32). These subtypes were (1) older age at onset and rapid disease progression, (2) young-onset PD and slow progression, (3) TD phenotype, and (4) PIGD phenotype. However, it is still not known how stable these subtypes are (33). Genetics and biomarkers can contribute significantly toward better subtyping of PD. While it is unlikely that any single marker will be able to define PD subtypes, biomarkers may assist in characterizing subtypes. As an example, it has been shown that the CSF level of α-synuclein was significantly lower in the non-tremor-dominant subtype of PD (34).

Our study's strength is the 3-year longitudinal follow-up of patients to assess the stability of their motor subtypes. While we recognize that other factors such as physiotherapy could affect subtype classification and stability, our sample is an early PD cohort, and most of the patients at this stage have not received active physiotherapy in our clinic setting. We also only assessed the MMSE data at baseline and will be following the cohort to determine if their cognitive status is affected by longer-term subtype stability.

In summary, we demonstrated that PD motor subtypes as defined by the MDS-UPDRS become unstable over time in the early course of the disease, with only 50–62% of patients remaining stable over 3 years. We have also shown that a higher TD/PIGD ratio and the inclusion of LEDD at baseline may improve the identification of TD patients who remain stable with a TD subtype at 3 years. These findings have implications for clinical studies of PD subtypes, the evaluation of biomarkers for subtypes, and the design of clinical trials. Further studies on larger populations to validate these findings are needed.

## Data Availability Statement

The raw data supporting the conclusions of this article will be made available by the authors, without undue reservation.

## Ethics Statement

The studies involving human participants were reviewed and approved by the Singapore Health Institutional Review Board. The patients/participants provided their written informed consent to participate in this study.

## Author Contributions

AK and SYEN: data analysis and manuscript writing. AW, NC, XC, DH, WL, H-LN, S-TC, SXMN, ZX, K-YT, W-LA, and E-KT: data collection and patient referral. LT: conception, design, and manuscript writing. All authors involved have read, and approved the submitted version.

## Funding

This study was supported by the Singapore Ministry of Health's National Medical Research Council under its Open Fund Large Collaborative Grant (MOH-OFLCG18May-0002).

## Conflict of Interest

The authors declare that the research was conducted in the absence of any commercial or financial relationships that could be construed as a potential conflict of interest.

## Publisher's Note

All claims expressed in this article are solely those of the authors and do not necessarily represent those of their affiliated organizations, or those of the publisher, the editors and the reviewers. Any product that may be evaluated in this article, or claim that may be made by its manufacturer, is not guaranteed or endorsed by the publisher.
